# An interactive game for rehabilitation based on real-time hand gesture recognition

**DOI:** 10.3389/fphys.2022.1028907

**Published:** 2022-10-26

**Authors:** Jiang Chen, Shuying Zhao, Huaning Meng, Xu Cheng, Wenjun Tan

**Affiliations:** ^1^ College of Information Science and Engineering, Northeastern University, Shenyang, China; ^2^ College of Economics and Management, Shenyang Agricultural University, Shenyang, China; ^3^ Key Laboratory of Intelligent Computing in Medical Image, Ministry of Education, Northeastern University, Shenyang, China

**Keywords:** hand gesture recognition, graph convolutional network, residual mechanism, rehabilitation, human–computer interaction

## Abstract

Currently, cardiovascular and cerebrovascular diseases have become serious global health problems related to their high incidence and fatality rate. Some patients with cardiovascular cerebro-cardiovascular diseases even may face motor or cognitive dysfunction after surgery. In recent years, human–computer interactive systems with artificial intelligence have become an important part of human well-being because they enable novel forms of rehabilitation therapies. We propose an interactive game utilizing real-time skeleton-based hand gesture recognition, which aims to assist rehabilitation exercises by improving the hand-eye coordination of the patients during a game-like experience. For this purpose, we propose a lightweight residual graph convolutional architecture for hand gesture recognition. Furthermore, we designed the whole system using the proposed gesture recognition module and some third-party modules. Finally, some participants were invited to test our system and most of them showed an improvement in their passing rate of the game during the test process.

## 1 Introduction

We have previously reported research in the field of tech-assisted rehabilitation (Liu et al., 2019; Tan et al., 2021; Sun and Wu, 2022). Cardiovascular and cerebrovascular diseases have become serious global health problems because of their high incidence and fatality rate. Cardiovascular disease is the leading cause of death, accounting for about 34% of all deaths worldwide (Anteneh et al., 2021), followed by stroke, a typical cerebrovascular disease, accounting for 11.6% (Kisa et al., 2021). Even after treatment, both cardiovascular and cerebrovascular disease may lead to motor or cognitive dysfunction, which needs a long period of rehabilitation (Berthier, 2005; Pattanshetty et al., 2015). Aerobic and strength training programs can improve cognitive performance even during the chronic stroke phase (Oberlin et al., 2017). For patients, a rehabilitation process combining motor and cognitive training has the potential to enhance their chance of recovery and rebuild their ability to take care of themselves.

In the last few years, an increasing trend in the innovation of rehabilitation methods using new technology to make rehabilitation processes more efficient has emerged. A good rehabilitation method should be user-friendly and interesting, so that patients may engage in the process and have a good time. Human–computer interactive systems free the patients from having to travel to rehabilitation clinics and offer them an opportunity to do these exercises at home. Because patients might perform the exercises casually or incorrectly in a home-based environment, many advances in technology have been leveraged to make the process more immersive, such as 3D gaming (Nasri et al., 2020) and virtual reality (VR) (Rincon et al., 2016).

This article proposes an interactive game controlled by 10 hand gestures for people, who suffer from limitations in daily life caused by aging or a health condition and thus want to strengthen their hand–eye coordination or do brain exercises. Our contributions can be summarized as follows:

• A rehabilitation game that leverages artificial intelligence (AI) (hand gesture recognition and character recognition).
• A network architecture for real-time skeleton-based hand gesture recognition.

The article is organized as follows: Section 2 reviews the state-of-the-art rehabilitation methods with technological assistance and hand gesture recognition approaches. Section 3 describes the details of the interactive game. This section also describes the network architecture and introduces the whole system we designed to keep the application more reliable. Section 4 presents more details about the experimental process and the results obtained. In Section 5, we report the study conclusions and provide future research directions.

## 2 Related work

### 2.1 Rehabilitation system

Conventional rehabilitation utilizes methods including the mini-mental state examination (MMSE), neurobehavioral cognitive status examination, Loewenstein occupation therapy cognitive assessment, and Wechsler memory scale to train and evaluate rehabilitation. Some of these use real cards to train the patients and often ask them to choose the correct card or sort them in order. These approaches require a long intervention cycle and are difficult for patients to maintain good exercise independence for a long time.

With the development of computer technology, the PC has become an auxiliary tool in cognitive rehabilitation training. In the beginning, it only used simple interactive logic to guide patients through rehabilitation (Hofmann et al., 1996a). In 1996, Hofmann’s team introduced computer graphics into cognitive training (Hofmann et al., 1996b), making the program more interactive with colored images. The rapid development of computer graphics and human–computer interactions has led to more visual and audio usage in rehabilitation systems. The newest rehabilitation methods leverage AI and VR technology. A system with AI can recognize a patient’s behavior, such as hand gestures through vision or sensor data, which makes human–computer interactions more natural and convenient. Relatively, a system with VR allows patients to do rehabilitation exercises in any location, with an insignificant difference from the real world.

### 2.2 Hand gesture recognition

According to the type of input, methods of hand gesture recognition can be classified into two categories: image-based methods and hand skeleton-based methods. The former adopts image sequences, whereas the latter uses sequences of hand joints as input. Compared with image-based methods, hand skeleton-based methods relieve the difficulty in recognizing a cluttered background, and requires lower computation cost, thus enabling real-time hand gesture recognition to be installed on small devices (Zhang et al., 2020). However, a skeleton-based approach needs additional tools to extract the hand skeleton accurately.

According to the method used to extract features, hand gesture recognition methods can also be classified into two categories: hand-crafted feature-based methods and end-to-end-based methods. The former encodes the input data into handcrafted features, such as joint angle similarities (Ohn-Bar and Trivedi, 2013) and time sequence similarity (Plouffe and Cretu, 2016). The former encodes the sequences of joints into embedding vectors and often feeds them into deep-learning architecture, like recurrent neural networks (RNNs) or convolutional neural networks (CNNs), to extract deep features. Graph convolutional network (GCN) also has an important role in skeleton-based hand gesture recognition. Many studies have demonstrated the effectiveness of GCN using the skeleton as a graph structure (Yan et al., 2018; Zhang et al., 2020).

## 3 Methods and materials

### 3.1 Graph convolutional network architecture

Because the input data were acquired as a sequence of 21 hand joints, which is a natural graph structure, we chose to work with a graph convolutional network. For each hand with *N* joints, we defined a skeleton graph G(V, E) , where $V=\left\{v_{i}|i=0,\;1,\ldots ,\;N-1\right\}$ is the set of nodes ( $v_{i}$ denotes the j -th joint) and $E=\left\{e_{i,j}|i,j\in V\right\}$ is the set of edges. In our system, we set N=21 .

**FIGURE 1:**
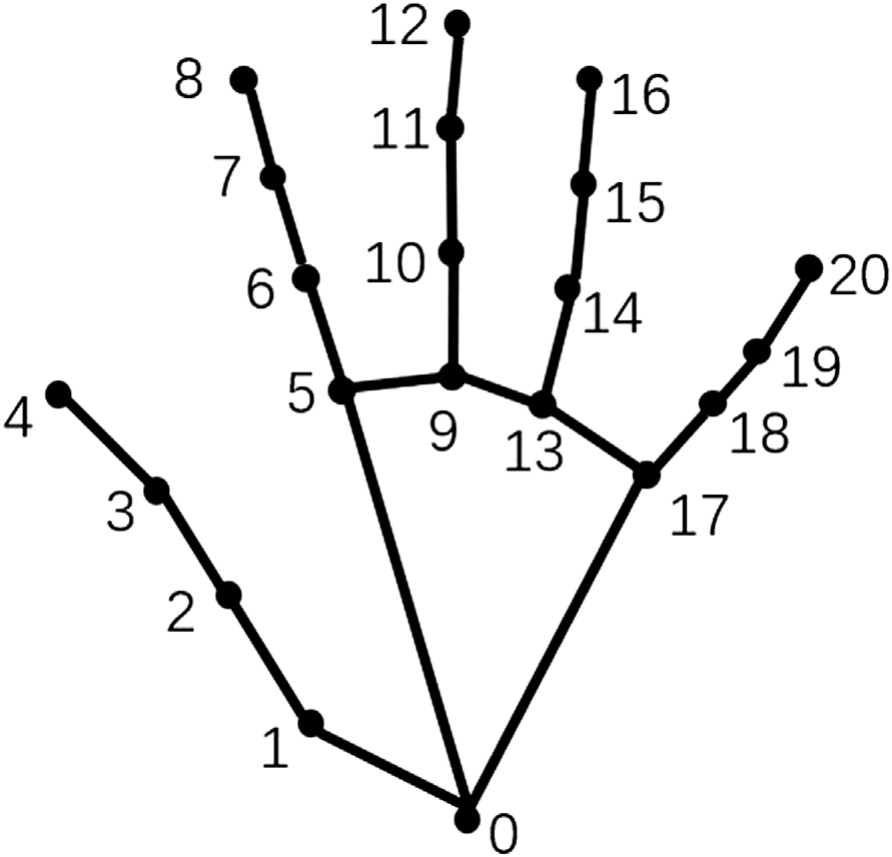
The hand skeleton graph. The nodes denote the hand joints and the edges denote the connections.

The topological relationship of nodes in the hand skeleton graph is illustrated in Figure 1. The edge-connecting nodes $v_{i}$ and $v_{j}$ mean node $v_{i}$ and $v_{j}$ have connected joints in the hand skeleton tree. It also means that nodes $v_{i}$ and $v_{j}$ are adjacent. The graph convolution propagation rule of the graph G can be described with the following formula:

$$F^{\left(l+1\right)}=\sigma \left(A^{s}W_{s}^{\left(l\right)}F^{\left(l\right)}\right),A^{s}=D_{s}^{-1/2}A^{s}D_{s}^{-1/2},$$ (1)

where $A^{s}$ is the normalized adjacent matrix of G , $W_{s}^{\left(l\right)}$ of the size $C_{out}\times C_{in}\times 1\times 1$ is a weight matrix to be trained (Cout and Cin are the numbers of input and output channels), $F^{\left(l\right)}$ denotes the output of the l -th layer, σ(·) is the activation function of the graph convolutional layer, and $D_{s}$ is the degree matrix of $A^{s}$ .

Before sending the data into the model, we preprocessed the data by setting the origin of the coordinates in the palm (midpoint between $v_{0}$ and $v_{9}$ ), making the data more evenly distributed. To prevent overfitting, the input hand data are randomly rotated as a data augmentation approach. The rotation angle θ∼N(0,π/10) .

**FIGURE 2:**
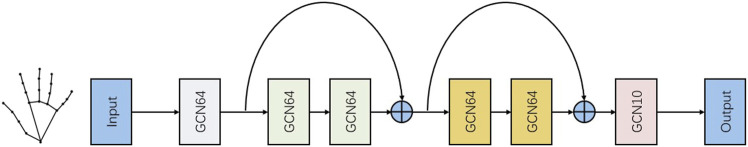
Illustration of our graph convolutional network architecture. The input is the coordinates of 21 hand joints. The two side paths that both bypass two 64-channel GCN, bring residual mechanism to our architecture.

Figure 2 illustrates our proposed neural network architecture for hand gesture recognition. In this model, we implemented one graph convolutional layer with 64 channels to transform the input 3-channel data into a 64-channel vector. This was followed by two graph convolutional units with the residual mechanism, which requires making a straight connection between the input and output of every unit (Figure 2). This is effective at resolving gradient disappearance. Each unit has two layers of graph convolution with 64 channels of output. Finally, we fed the feature vector into a graph convolutional layer with 10 channels to match the number of gesture categories. We used softmax to calculate the probability of each class. The architecture described is shown in Figure 2.

### 3.2 Rehabilitation game experience

We created an interactive game controlled by hand gestures. This game utilizes a camera to recognize the hand behavior of the player and motivate the player to do air writing. It provides a hand–brain combined exercise and facilitates the rehabilitation process.

**FIGURE 3:**
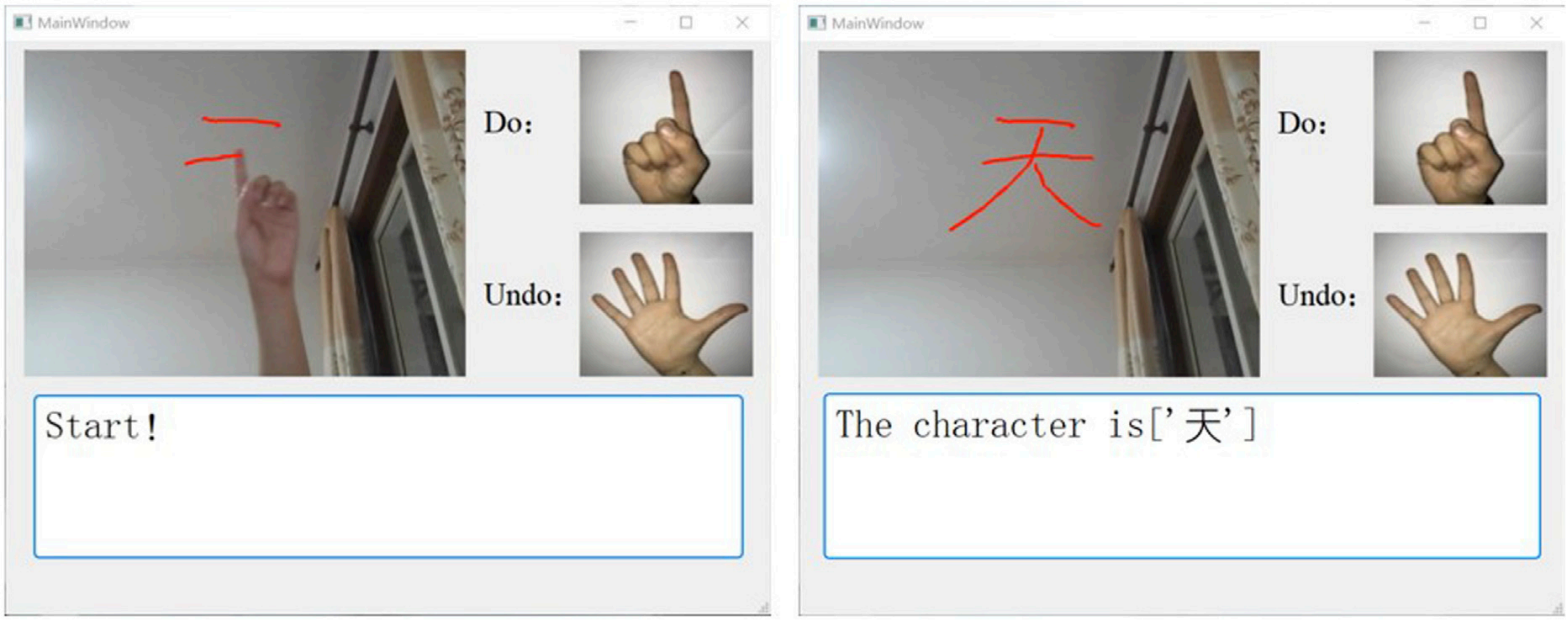
Illustration of one example of the rehabilitation game experience. **(A)** shows the process of writing in the air. **(B)** shows the result of the character recognition.

At the beginning of the game, two hand gestures were given by the computer to control an invisible pen to draw in the air (by generating a line or undoing a line). The player is required to control the pen correctly to complete a Chinese character. The system displays the player’s finger movements as they write and show the recognition result after they finish handwriting (Figure 3).

### 3.3 Design of the rehabilitation system

**FIGURE 4:**
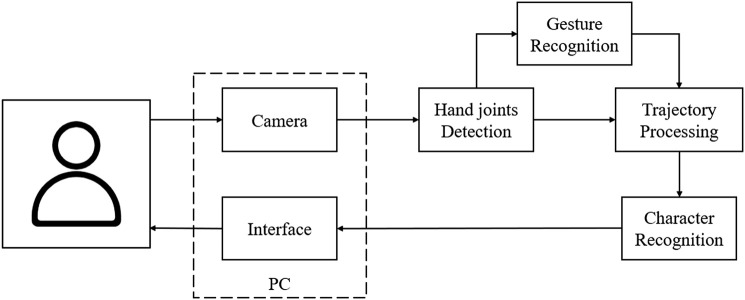
Designed diagram of the proposed system. Each block denotes a single module in our system. The two modules in the dotted box belong to a PC.

The system obtains visual images from the computer camera and communicates with users through an interface. The backend of the system is composed of a hand joint detection module, a gesture recognition module, a trajectory processing module, and a character recognition module. The visual image is first sent to the hand joint detection module and transformed into 3D coordinates of 21 hand joints. Then, the gesture recognition module identifies the specific gesture category. The trajectory processing module controls the generation of handwriting according to the hand behavior of the player. After that, it also performs Bezier smoothing and then passes the image with the completed character to the next module. Finally, the character recognition module recognizes the image with the character and delivers them back to the interactive interface, which will guide the player to interact further *via* the camera. The system described is shown in Figure 4.

## 4 Experiments and results

**FIGURE 5:**
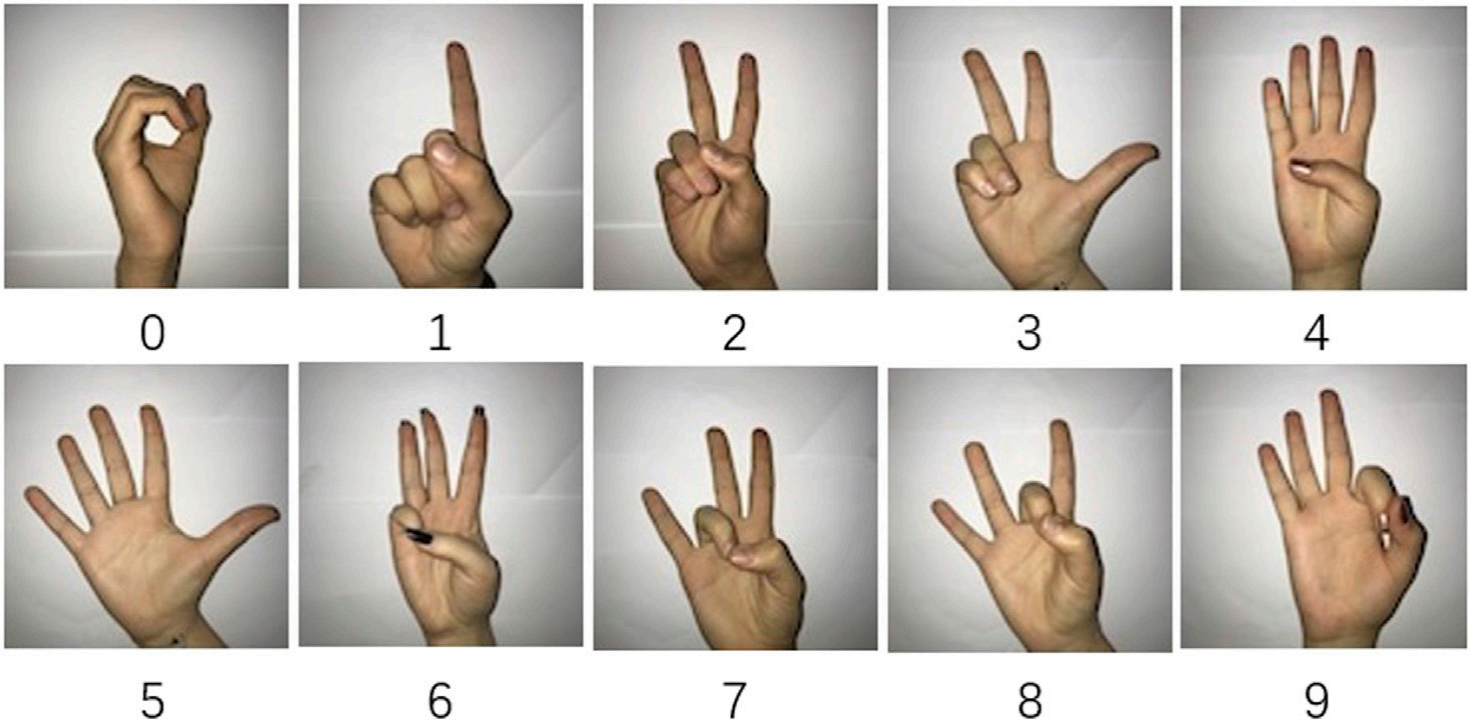
Samples of the dataset. It consists of 2062 RGB pictures labeled by 10 gesture classes (from gesture “0” to “9”).

In this section, we evaluate the performance of our model for skeleton-based action recognition. We used the American Sign Language Digits Dataset (Mavi, 2020) in our experiments. This consists of 2062 RGB pictures labeled by 10 gesture classes (from gesture “0” to “9”). These pictures are taken from 218 different students, making the hand features to be learned more diverse. We implemented a 5-fold cross-validation technique in our experiments, and they were conducted *via* a GeForce GTX 1650 GPU. Some examples of the dataset images are shown in Figure 5.

We used cross-entropy as a loss function. The models were learned using a stochastic gradient descent with a learning rate of 0.001. We decayed the learning rate to 0.9 after every 30 epochs. To restrain extreme weights, we implemented L1 regularization with a parameter of 5e-3 and a weight decay (L2 regularization) with a parameter of 1e-4.

**FIGURE 6:**
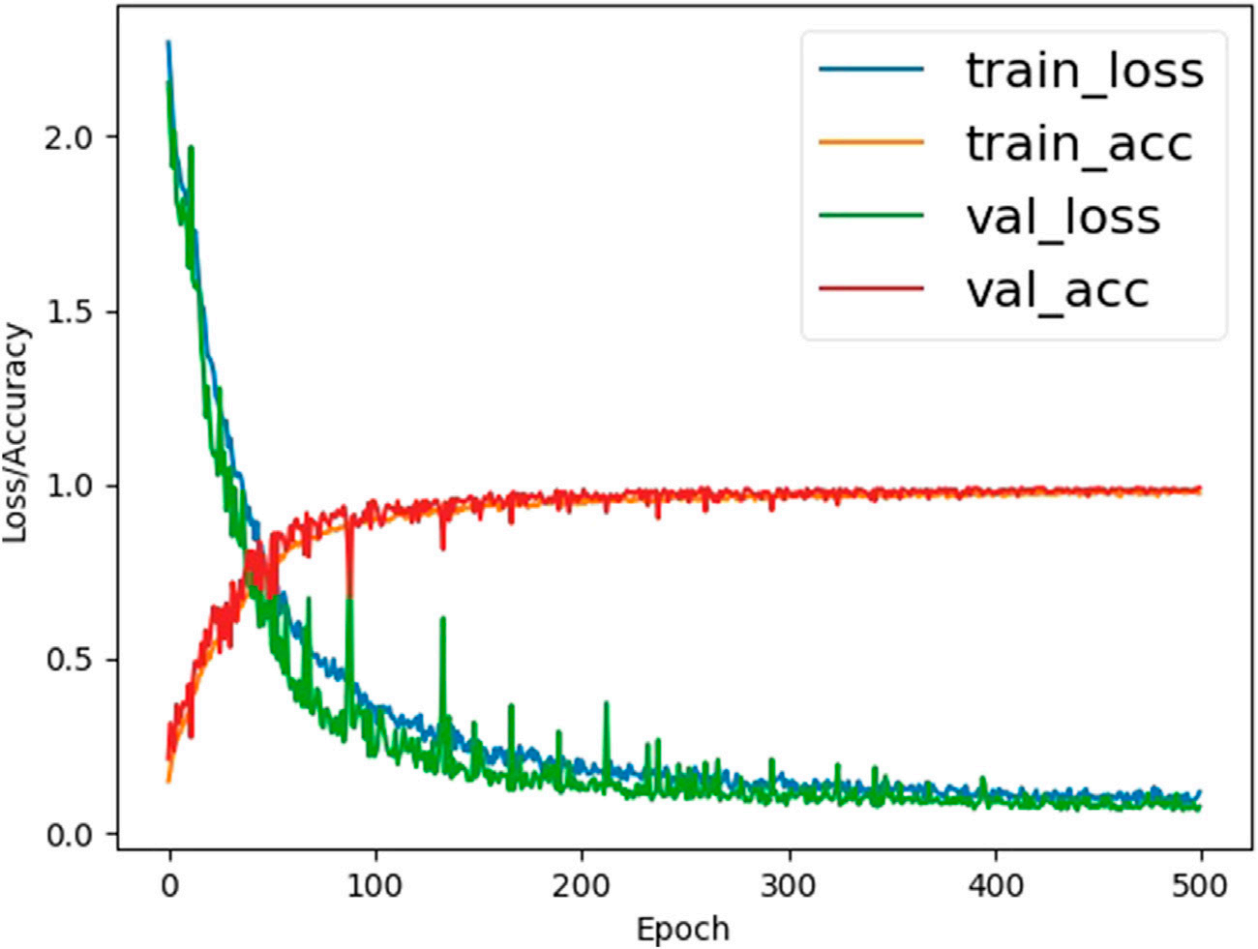
Loss and accuracy graph of the training process. The horizontal axis represents epoch and the vertical axis represents loss and accuracy value.

**FIGURE 7:**
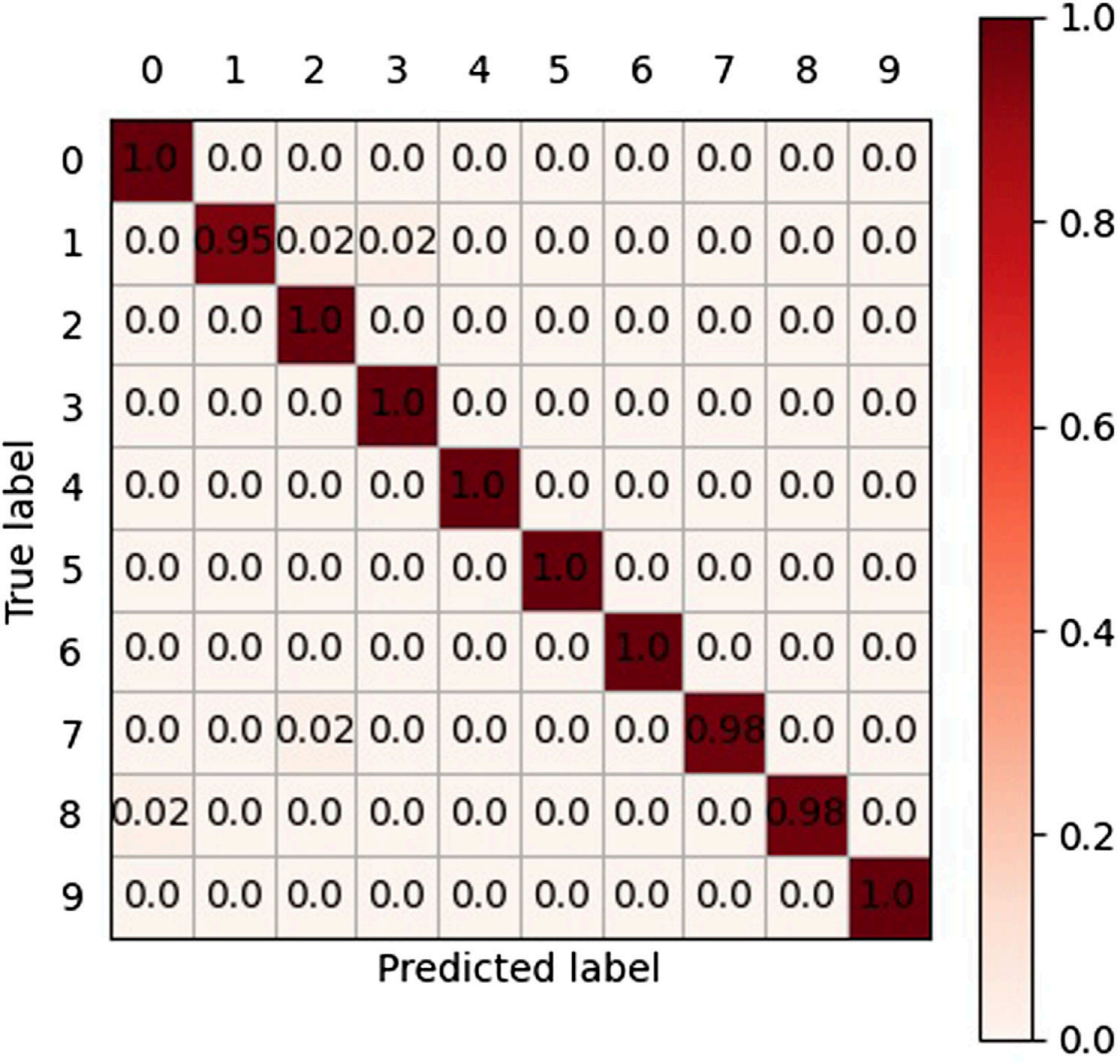
Chaotic matrix of all the samples in the test experiment. The diagonal values of the matrix are the rates of correct classifications, while the non-diagonal values are the rates of wrong classifications.

Our cross-validation strategy is as follows: in each round, we used 4 folds of data for training and 1 fold of data for validation. This process lasts for 5 rounds during each epoch to ensure every fold is chosen as validation data at least once. In this case, the loss and accuracy of one epoch are calculated as the average loss and accuracy of 5 rounds. The model is trained for 500 epochs. During the training period, both the train loss and validation loss decrease continuously, indicating the network is trained properly. After about 200 epochs, the loss value becomes stable. The validation accuracy peaked at 99.03%, with an insignificant difference in training accuracy (Figure 6). As shown in the confusion matrix (Figure 7) we recorded, the system was reasonably capable of classifying different hand gestures from “0” to “9.”

An ablation experiment was performed to better show the effect of the residual mechanism in our architecture (Table 1). The difference between the Naive model and Residual model was whether there was a forward path from the input to the output of each graph convolution unit. The FPS indicates how many frames can be processed per second. According to the results, the residual module improved the recognition accuracy by 1% while maintaining a fast processing speed and a small size. The architecture is relatively lightweight because the prediction cost of each gesture is about 6 ms and the size of the model is only 72.5 KB. This enables the game to support many small devices, which usually do not have enough memory, and to calculate the performance required to support an AI application.

**TABLE 1:** Ablation study.

Methods	Acc (%)	FPS (/s)	Size (KB)
Naive model	98.03	178.51	72.50
+ Residual	99.03	178.32	72.50

Comparisons have been made between our model and other state-of-the-art models (Table 2). The first CNN (Mavi, 2020) is a convolution network with more convolution layers than MVGG-5 and less than MVGG-9 architecture. MobileNetV2 (Dayal et al., 2021) is a state-of-the-art lightweight CNN. The second CNN (Dayal et al., 2021) is a memory-efficient convolution network based on a bottleneck module, specifically designed for edge computing systems. According to Table 2, using the same dataset, the accuracy of our model is comparable to state-of-the-art models, although our model has a significant advantage in model size.

**TABLE 2:** Comparisons with state-of-the-art models.

Model	Acc (%)	Size
CNN (Mavi, 2020)	98.00	17.2 MB
MobileNetV2 (Dayal et al., 2021)	98.50	8.5 MB
CNN (Dayal et al., 2021)	99.10	4 MB
Ours	99.03	72.5 KB

The system has been tested by four participants. All subjects were given instructions before playing and were allowed to play several rounds to become familiar with the rules. The participants were required to write 12 characters correctly in each round of the test by using their hand behavior in front of the system camera. They needed to write the indicated character as clearly as possible to ensure it could be recognized by the word recognition module. There were several minutes of rest between every two rounds. Their passing rates in each round and their opinions on the game experience are recorded in Table 3. According to the results, most of the subjects showed an improvement in the passing rate during five rounds of testing. However, it seems to be hard to reach a passing rate of 100% because of difficulty in writing in the air and some problems in the third-party word recognition module, which can be substituted and improved. Despite this, all the subjects agreed that the game experience was smooth and interesting.

**TABLE 3:** Passing rates and opinions of participants.

	Passing rate (%)					Opinion
	Round 1	Round 2	Round 3	Round 4	Round 5	
Subject 1	0.58	0.58	0.75	0.67	0.75	Handwriting in the air is difficult
Subject 2	0.50	0.50	0.58	0.58	0.50	The word recognition seems not to work well
Subject 3	0.75	0.67	0.67	0.75	0.92	It is difficult to control the pen trajectory as I want
Subject 4	0.58	0.50	0.75	0.67	0.67	It is interesting

## 5 Conclusion

This work proposes an interactive game and a human–computer interaction system for rehabilitation usage. We also propose a new residual graph convolution structure for skeleton-based gesture recognition. The model was trained for the recognition of 10 static hand gestures and was evaluated after the experiments. The system achieved 99.03% validation accuracy and maintained a relatively small size of 72.5 KB. The experimental results demonstrated that our model was sufficiently accurate as a gesture recognition system and that the game has the potential to be extended for rehabilitation usage. Four participants were invited to test this interactive game, and most subjects showed an improvement and interest during the test.

We should consider more motivational rewards and better hand gesture recognition models for the rehabilitation game. Additionally, the interactive process should be enhanced to be more smooth and interesting.

## Data Availability

Publicly available datasets were analyzed in this study. These data can be found at: https://github.com/ardamavi/Sign-Language-Digits-Dataset/.
